# Repurposing chlorpromazine in the treatment of glioblastoma multiforme: analysis of literature and forthcoming steps

**DOI:** 10.1186/s13046-020-1534-z

**Published:** 2020-01-31

**Authors:** Claudia Abbruzzese, Silvia Matteoni, Michele Persico, Veronica Villani, Marco G. Paggi

**Affiliations:** 1 Cellular Networks and Molecular Therapeutic Targets, Proteomics Unit, IRCCS - Regina Elena National Cancer Institute, Via Elio Chianesi 53, 00144 Rome, Italy; 2 Neuro-Oncology, IRCCS - Regina Elena National Cancer Institute, Rome, Italy

**Keywords:** Brain tumors, Antipsychotic drugs, Drug repositioning, Signal transduction, Clinical trial

## Abstract

**Background:**

Glioblastoma multiforme is a CNS cancer characterized by diffuse infiltrative growth, aggressive clinical behavior and very poor prognosis. The state-of-art clinical approach to this disease consists of surgical resection followed by radiotherapy plus concurrent and adjuvant chemotherapy with temozolomide. Tumor recurrence occurs in virtually all cases, therefore, despite any treatment, the median survival is very low (14.6 months), which makes the approach to these patients a challenging clinical issue.

**Main body:**

The escalating costs and times required for new medications to reach the bedside make repurposing or repositioning of old drugs, when scientific bases allow their use in other pathologies, an appealing strategy. Here, we analyze a number of literature data concerning the antipsychotic chlorpromazine, the founder of the phenothiazines class of drugs, a medication widely used in the clinics for approximately 60 years. The drug exerts its effects on psychiatric patients by interfering with the dopamine receptor D_2_, although more recent pharmacodynamics studies ascribe chlorpromazine a series of biological effects on cancer cells, all converging in hindering also glioblastoma survival capabilities.

**Short conclusions:**

On these bases, and assisted by the information on the well-established chlorpromazine toxicity and dosage in humans, we designed a Phase II clinical trial involving the combination of chlorpromazine with the standard treatment, temozolomide, in the adjuvant phase of the therapeutic protocol. Patients displaying hypo-methylation of the MGMT gene, and thus intrinsically resistant to temozolomide, will be enrolled. The endpoints of this study are the analysis of toxicity and clinical activity, as evaluated in terms of Progression-Free Survival, of the association of chlorpromazine with the first-line treatment for this very serious form of cancer.

## Background

Glioblastoma multiforme (GBM), the most frequent and lethal CNS malignant tumor, is characterized by an exceptionally dismal prognosis, with a median patient survival time of 14.6 months, which makes GBM patient management an unmet clinical need. The current treatment in newly diagnosed patients consists in maximal well-tolerated surgical resection followed by radiotherapy plus concurrent and adjuvant chemotherapy using the alkylating drug temozolomide (TMZ). This therapeutic scheme has substantially remained unchanged for 15 years and utilizes a single anticancer compound. Despite the identification of targetable driver genes in GBM, its extreme intra-tumor heterogeneity and the consequent plasticity make it resistant toward targeted therapies. Considerable and continuous efforts in searching for novel pharmacological approaches are strongly encouraged to fight against such a grave condition.

According to the current rules, by either the Food and Drug Administration (FDA) or the European Medicines Agency (EMA), potentially useful new drugs must travel a “long and winding road” in order to effectively reach the bedside. Therefore, rational and motivated repurposing of clinically well-characterized drugs can represent an attractive alternative, making the development of new therapies possible by using old compounds whose clinical administration is associated with lower risks, shorter bench-to-bedside timelines and lower costs.

## Main text

Chlorpromazine (CPZ, Largactil, Thorazine), the progenitor of the tricyclic antipsychotic compounds phenothiazines, has been effectively and safely employed for over half a century in the treatment of psychiatric disorders. Its role in these pathologies is essentially attributed to the ability to act as a potent antagonist of the dopamine receptor D_2_ (DRD2) [1]. Besides this well-established pharmacological mechanism of action, CPZ caught our attention due to a remarkable series of biomolecular effects observed in cancer cells that are well described in the scientific literature and that we would like to briefly bring to the reader’s attention.

### Hindrance of Cancer cell growth

CPZ is cytotoxic for many cancer cells in vitro, especially malignant gliomas [2, 3]. In non-neoplastic cells, CPZ shows a reversible cytostatic activity, with the exception of fibroblasts, where a toxic effect results detectable [2].

### Nuclear aberrations

CPZ induces nuclear fragmentation in vitro, which can be responsible for the mitotic catastrophe described in cancer cells under the effect of this drug. In this setting, importance is given to the ability of CPZ to inhibit the activity of the mitotic kinesin KSP/Eg5 [4], thus hampering the correct spindle formation and chromosomal distribution between the daughter cells.

### Inhibition of the PI3K/mTOR axis

CPZ is proficient in inhibiting the AKT/mTOR axis in malignant glioma cells [5], a pathway that plays a pivotal role in regulating cell metabolism and ATP homeostasis.

### Induction of autophagy

As a direct consequence of the ability of CPZ to decrease mTOR activity, cells exposed to this compound appear to activate an autophagic program [5] that, while it can represent a survival-oriented mechanism in normal cells under energy deprivation, it could evolve toward cytotoxicity in cancer cells, being these latter already under stressed conditions or demanding bioenergetics requirements.

### Inhibition of glutamate receptors

Interestingly, CPZ is also known to inhibit the AMPA glutamate receptor [6], which has been very recently recognized as highly expressed in GBM and fundamental in driving its growth and progression [7, 8]. Additionally, CPZ appears to also be effective in inhibiting the NMDA glutamate receptor [6], described as essential for the nesting and growth of brain metastases from breast cancer [9].

### Inhibition of dopamine D_2_ receptor

The well-known effect of CPZ as an antagonist of the dopamine receptor D_2_ plays a further significant role in reducing GBM metabolism, signaling and plasticity [10].

**Fig. 1:**
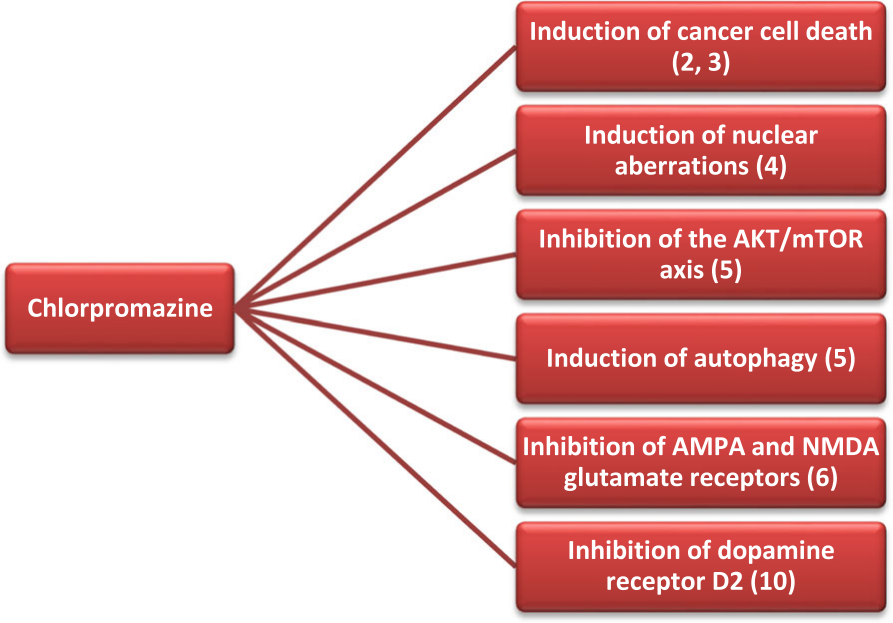
Multiple effects of CPZ on GBM cells. The biological processes where CPZ exerts a detrimental role in GBM growth and survival parameters are represented. The associated references are also indicated

All these effects of CPZ on cancer cells and their homeostasis, achieved by exposing cancer cells to the drug in the low micromolar range and for short times, are summarized in Fig. 1.

CPZ is employed in the clinical treatment of psychiatric disorders also for chronic administration, according to patient needs. Treatment exposes the patient to sometimes severe, but dose-dependent, reversible and manageable side effects. Thus, such adverse reactions should not hinder GBM patient treatment, especially if the poor prognosis attainable with the first-line, approved therapeutic scheme is considered. Not least, the drug is freely permeable through the Blood-Brain Barrier.

In our laboratory, we are actively pursuing an up-to-date evaluation of the pharmacodynamic properties of CPZ on GBM cells, by employing proteomic approaches aiming to identify a more exhaustive spectrum of its targets, as well as its overall effects on the fundamental signal transduction pathways that are vital for cancer cell survival.

## Conclusions

Such a noticeable and multifaceted series of effects of CPZ on malignant glioma cells in vitro motivated us to design a clinical trial employing this compound in GBM patients. Given that CPZ has been used in the clinics since the ‘50s, all the data regarding its dosage range and toxicity as a single drug are well established. Such knowledge allowed us to bypass the Phase I experimentation and to plan directly a Phase II clinical trial. The experimental protocol involves the combination of CPZ with the standard treatment with TMZ in the adjuvant phase of the first-line therapeutic protocol (after radio-chemotherapy, TMZ for consecutive 5 out of 28 days, at a dose of 150–200 mg/sq. m, for six cycles). CPZ will be administered orally at a dosage of 50 mg/day (from day 1 to 28) during each of the six cycles of the adjuvant treatment with TMZ. The primary endpoint of this study is the evaluation of the toxicity of the combined treatment. The secondary endpoint is the evaluation of the clinical activity, in terms of Progression-Free Survival (PFS), of this drug association. Only patients carrying hypo-methylation of the MGMT gene, i.e. those with worse prognosis due to their intrinsic resistance to TMZ, will be enrolled. Despite being aware of the relevance of patient sex in GBM incidence, clinical course and, not last, therapeutic toxicity, we chose to enroll both male and female patients.

This clinical trial was submitted to the Institutional Ethical Committee (Comitato Etico Centrale IRCCS - Sezione IFO-Fondazione Bietti, Rome, Italy) and was approved on 6th September 2019 (EudraCT # 2019–001988-75; ClinicalTrials.gov Identifier: NCT04224441).

We consider the possibility to directly perform a Phase II clinical trial as a rational and ethical option to be pursued, dealing with patients for which there are very limited therapeutic options available. If successful, the use of a repurposed compound will help in decreasing expenses and development time for a drug in order to effectively reach the bedside.

## Data Availability

Not applicable.

## Abbreviations

CPZ: Chlorpromazine
DRD2: Dopamine receptor D_2_
EMA: European Medicines Agency
FDA: Food and Drug Administration
GBM: Glioblastoma multiforme
PFS: Progression-Free Survival
TMZ: Temozolomide
